# Cryoballoon Versus Radiofrequency Ablation for Persistent Atrial Fibrillation: Meta‐Analysis of Randomized Trials

**DOI:** 10.1111/pace.70350

**Published:** 2026-07-03

**Authors:** Harroop Bola, Aksaan Arif, Joshua J. Hon, Gayathri Giritharan, Prachur Khandelwal, Rodrick Avakian, Amar Rai, Kiran Haresh Kumar Patel, Fu Siong Ng

**Affiliations:** ^1^ Faculty of Medicine Imperial College London, England London UK; ^2^ London Northwest Healthcare NHS Trust, England London UK; ^3^ Department of Internal Medicine University of Pennsylvania Philadelphia Pennsylvania USA; ^4^ Chelsea and Westminster Hospital NHS Foundation Trust, England London UK; ^5^ National Heart and Lung Institute Imperial College London, England London UK; ^6^ Department of Cardiology Imperial College Healthcare NHS Trust, England London UK

**Keywords:** catheter ablation, cryoballoon ablation, radiofrequency ablation, persistent atrial fibrillation

## Abstract

**Introduction:**

Pulmonary vein isolation (PVI) alone achieves modest arrhythmia freedom in persistent atrial fibrillation (PeAF). Cryoballoon ablation (CBA) offers a single‐shot alternative to radiofrequency ablation (RFA) that streamlines procedural workflow. We aimed to quantify the clinical efficacy, procedural efficiency, and safety of CBA versus RFA through a meta‐analysis of randomized controlled trials (RCTs).

**Methods and Results:**

Systematic review of PubMed, Embase, and Scopus identified 5 RCTs enrolling 1,091 patients (515 CBA, 576 RFA). The primary endpoint was 12‐month freedom from any atrial arrhythmia, with isolated AF, and atrial tachycardia/flutter evaluated as secondary endpoints. Random‐effects models generated relative risks (RRs) and mean differences (MDs) with 95% confidence intervals (CI). Time‐to‐event data were pooled using a generic inverse‐variance approach to derive hazard ratios (HR). CBA and RFA demonstrated equivalent freedom from any arrhythmia (RR 0.97, 95% CI 0.81–1.15; *p* = 0.71). Time‐to‐event analysis corroborated a comparable recurrence hazard (HR 0.91, 95% CI 0.67–1.24; *p* = 0.56; *I* ^2^ = 0%). Repeat ablation rates were identical (RR 0.93; *p* = 0.50). CBA significantly reduced procedural time (MD −45.37 min; p < 0.0001), while major complications were comparable (1.5% vs. 2.7%; *p* = 0.18). Trial sequential analysis indicated that further randomization is unlikely to demonstrate superiority.

**Conclusion:**

In PeAF, CBA and RFA achieve equivalent 12‐month arrhythmia‐free survival and comparable repeat‐ablation rates, while CBA confers a highly significant reduction in procedural time. However, reliance on 12‐month follow‐up and heterogeneous rhythm surveillance protocols may limit assessments of true long‐term durability.

## 1 Introduction

Pulmonary vein isolation (PVI) remains the mainstay of percutaneous catheter‐based ablation for atrial fibrillation [1] (AF), yet its efficacy as a standalone strategy in persistent AF (PeAF) is modest, with contemporary trials reporting single‐procedure arrhythmia‐free survival rates of only 30%–50% at 12 months [2, 3]. In PeAF cohorts, PVI is comparatively more challenging than in paroxysmal AF and exhibits higher rates of PV reconnection that limit long‐term durability^4^^–^ 6. Cryoballoon ablation (CBA) offers a single‐shot alternative to point‐by‐point radiofrequency ablation (RFA), and landmark trials such as FIRE AND ICE and CIRCA‐DOSE established that CBA is noninferior to RFA for rhythm outcomes while shortening procedure times [7, 8]. Recent PeAF trials have extended this signal, showing similar 1‐year efficacy with CBA and RFA but consistently greater procedural efficiency with CBA [9, 10, 11, 12, 13].

Comparative evidence evaluating CBA versus RFA in PeAF has historically been limited by reliance on observational, single‐center, or mixed‐phenotype data. Consequently, prior meta‐analyses have been vulnerable to confounding and selection bias, precluding definitive conclusions regarding relative efficacy and safety [14]. To address these methodological limitations, we conducted a systematic review and meta‐analysis restricted exclusively to randomized controlled trials (RCTs) comparing CBA and RFA for de *novo* PeAF ablation. The primary objective of this study was to evaluate 12‐month rhythm outcomes, procedural efficiency, and safety profiles to clarify the comparative clinical utility of these thermal modalities.

## 2 Methods

This systematic review and meta‐analysis were conducted in accordance with the Preferred Reporting Items for Systematic Reviews and Meta‐Analyses (PRISMA) 2020 guidelines and the methodological framework outlined in the Cochrane Handbook for Systematic Reviews of Interventions [15, 16]. The study protocol was prospectively registered with the International Prospective Register of Systematic Reviews (PROSPERO; registration number CRD420251142732) prior to initiation of the literature search and data extraction.

### 2.1 Literature Search Strategy

A comprehensive, systematic literature search was performed across PubMed, Embase, and Scopus from database inception through December 2025. The full search strategy is provided in the Supplementary Appendix. No restrictions were applied with respect to language, publication date, or geographical location.

### 2.2 Eligibility Criteria

Study eligibility was formulated according to the PICOS framework. Inclusion was strictly limited to parallel‐group RCTs, published as peer‐reviewed full‐text articles, that enrolled adult patients (≥18 years) with PeAF undergoing first‐time catheter ablation per contemporary consensus definitions. For the procedural modalities, trials were required to directly compare CBA (any commercially available system) against RFA (any catheter generation, including irrigated‐tip and contact‐force sensing systems) for PVI. Furthermore, studies had to report at least one prespecified efficacy, procedural, or safety endpoint with extractable quantitative data for both study arms, including a minimum follow‐up duration of 12 months for efficacy outcomes. We explicitly excluded studies restricted to paroxysmal AF, mixed AF populations lacking extractable PeAF subgroup data, repeat ablation cohorts, and pediatric populations. Trials employing non‐standard technologies, such as focal cryoablation, pulsed‐field ablation (PFA), laser balloon, or surgical/hybrid approaches, were also excluded.

### 2.3 Outcome Measures

#### 2.3.1 Primary Efficacy Endpoints

The primary efficacy endpoint was prespecified as any atrial arrhythmia recurrence at 12 months (a composite of AF, atrial tachycardia (AT), or atrial flutter (AFL) ≥30 s post‐blanking). Secondary efficacy endpoints included isolated AF recurrence at 12 months (AF episodes ≥30 s beyond a 90‐day blanking period) and AT/AFL recurrence at 12 months (organized atrial tachyarrhythmias ≥30 s post‐blanking). Prespecified procedural endpoints included total procedure duration, fluoroscopy time, and requirement for repeat ablation. The primary safety endpoint was the composite incidence of any procedure‐related complication within 30 days of ablation or during follow‐up. Secondary safety endpoints comprised phrenic nerve palsy (PNP), vascular access site complications, and pericardial effusion.

### 2.4 Study Selection and Data Extraction

All retrieved records were imported into Covidence (Veritas Health Innovation, Melbourne, Australia) for deduplication and screening. Following removal of duplicates (*n* = 1,349), titles and abstracts of 11,457 records were independently screened by at least two reviewers (A.A., G.G., J.J., R.A.). Full‐text review was conducted for 40 studies, of which 5 RCTs met all eligibility criteria and were included in the final quantitative synthesis. Disagreements were resolved through consensus, with adjudication by a third reviewer (H.B.) where necessary. Data extraction was performed independently and in duplicate using a standardized electronic form, capturing study characteristics, patient demographics, procedural details, and outcomes. For multi‐arm trials incorporating variations of the same modality (e.g., standard vs. extended CB per EXCAPE‐AF) [13], only the standard arm was included to preserve clinical homogeneity and avoid unit‐of‐analysis errors. Missing data were not imputed.

### 2.5 Quality Assessment and Risk of Bias

Risk of bias was independently assessed by two reviewers (A.A., G.G., J.J., R.A.) using the revised Cochrane Risk of Bias tool for randomized trials (RoB‐2) [17] tool across five domains: randomization, deviations from intended interventions, missing outcome data, outcome measurement, and selective reporting. Given the inherently non‐blinded nature of catheter ablation procedures, emphasis was placed on follow‐up completeness and objectivity of outcome ascertainment. Disagreements were resolved by consensus, with third‐reviewer adjudication (H.B.) where required.

### 2.6 Statistical Analysis

All analyses were performed using R statistical software version 4.5.2 (R Foundation for Statistical Computing, Vienna, Austria) with the *meta* package (version 7.0‐0). For dichotomous outcomes, relative risks (RRs) with 95% confidence intervals (CIs) were calculated using the Mantel–Haenszel method. For continuous outcomes, mean differences (MDs) with 95% CIs were estimated using the inverse‐variance method. Statistical significance was defined as *P* ≤ 0.05. Between‐study heterogeneity was assessed using the Cochran Q statistic (*p* < 0.10 indicating significant heterogeneity) and the *I* ^2^ statistic, interpreted according to Cochrane thresholds [16]. A random‐effects model was prespecified for all analyses to account for anticipated clinical and methodological heterogeneity. For clinically significant dichotomous outcomes, trial sequential analysis (TSA) was performed to evaluate the risk of random error and determine the diversity‐adjusted required information size, assuming a conservative 20% relative risk reduction [18].

### 2.7 Time‐to‐Event Analysis

To account for the temporal distribution of arrhythmia recurrence and evaluate treatment durability, a secondary time‐to‐event meta‐analysis was performed. Log‐transformed hazard ratios (HR) were pooled using a random‐effects generic inverse‐variance model. Between‐study variance was estimated using restricted maximum likelihood, aligning with current Cochrane recommendations. Statistical significance was defined as a two‐sided *p* value ≤0.05.

### 2.8 Publication Bias and Sensitivity Analyses

Publication bias was assessed by visual inspection of funnel plots. Formal statistical testing (e.g. Egger's regression) and meta‐regression was not performed due to an insufficient number of included studies (*n* = 5; minimum 10 required). Sensitivity analyses were conducted using a leave‐one‐out approach to assess the robustness of pooled estimates.

Results were presented using forest plots displaying study‐level and pooled effect estimates with corresponding 95% CIs.

## 3 Results

The initial systematic search yielded 12,806 records. Following the removal of 1349 duplicates and preliminary screening of 11,457 titles and abstracts, 40 full‐text articles were assessed for rigorous eligibility. Ultimately, 35 full‐text records were excluded, and 5 RCTs enrolling a total of 1091 patients were identified for inclusion in the final quantitative synthesis [9, 10, 11, 12, 13]. The complete study selection process is detailed in the PRISMA flow diagram (Figure 1).

**FIGURE 1:**
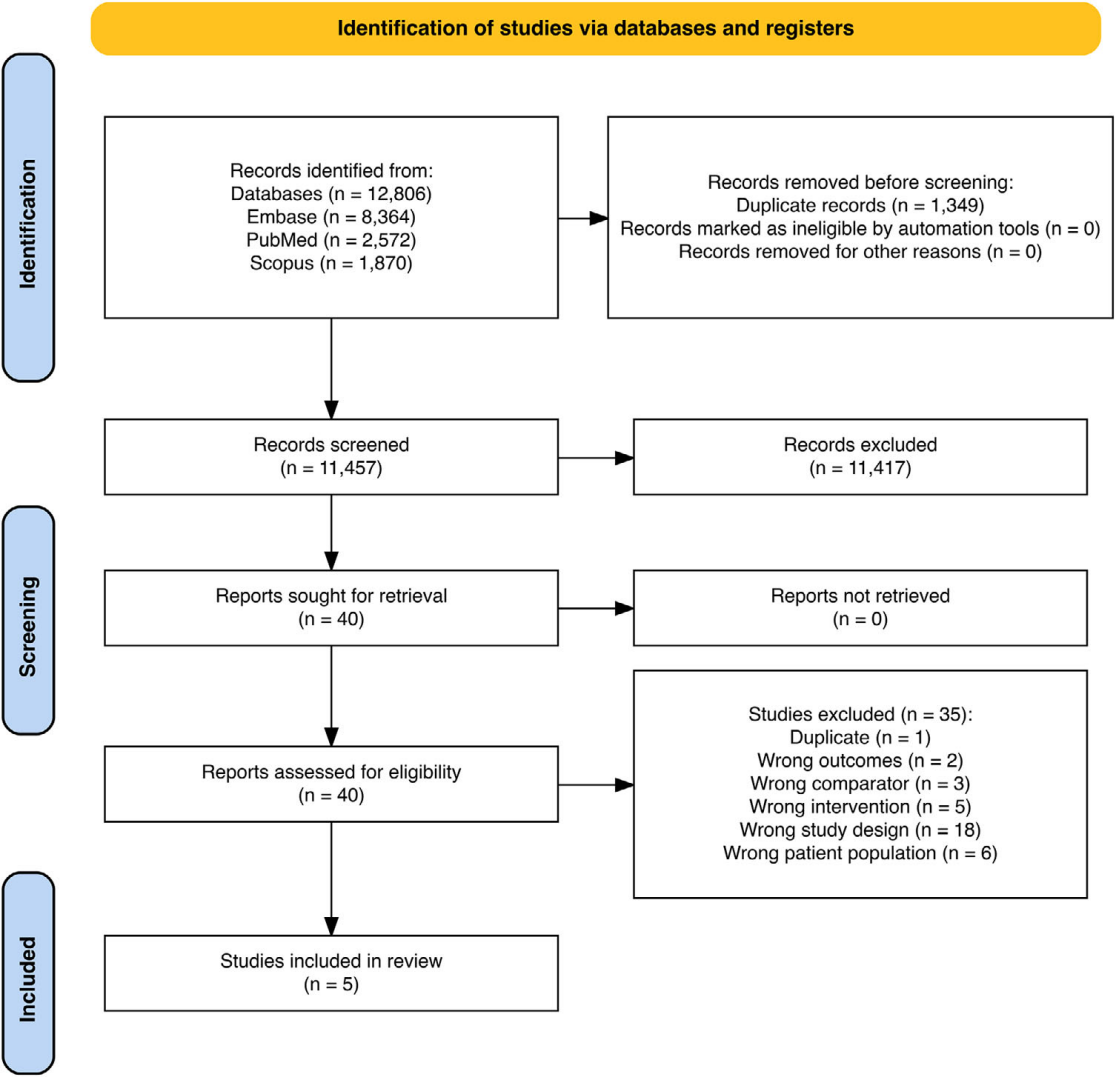
PRISMA flowchart. [Colour figure can be viewed at wileyonlinelibrary.com]

These trials consisted of 1 single‐center and 4 multicenter investigations, with all studies directly comparing CBA against RFA in patients with PeAF. Procedurally, the CBA cohorts utilized the second‐ through fourth‐generation Arctic Front Advance system (Medtronic), while the RFA cohorts uniformly employed contact‐force‐sensing irrigated ablation catheters.

### 3.1 Study Characteristics

The pooled population comprised 1,091 patients: 515 assigned to CBA and 576 to RFA. Among these, 829 (76.0%) were male, with a mean age of 64.4 years, mean left atrial diameter of 42.7 mm, and mean duration of known PeAF of 18.7 months prior to ablation. Baseline characteristics and trial designs are summarized in Table 1 and Table S2.

**TABLE 1:** Characteristics of included RCTs.

First author, year (study acronym or registry)	Country	No. Patients	Cryoballoon Catheter	Radiofrequency Catheter	Follow‐up, mo	Rhythm Monitoring Protocol
Multicenter NO‐PERSAF [9]	Norway	101	Arctic Front Advance, Medtronic (Second Generation)	Contact‐force‐sensing irrigated ablation catheter	12	Scheduled: Outpatient clinic evaluations accompanied by 7‐day ambulatory ECG monitoring at 3, 6, and 12 months after ablation.
Single center [10]	Kazakhstan	100	Arctic Front Advance, Medtronic (Second Generation)	Contact‐force‐sensing irrigated ablation catheter	36	Scheduled: Diagnostic evaluation of implanted electrocardiogram loop recorders at 3, 6, 9, 12, 18, 24, and 36 months.
						Adjunctive: Continuous automated rhythm surveillance via the implanted device throughout the follow‐up period.
Multicenter [11]	Greece	199	Arctic Front Advance, Medtronic (Second Generation)	Contact‐force‐sensing irrigated ablation catheter	12	Scheduled: 24 h Holter monitoring at 1, 3, 6, 9, and 12 months.
						Adjunctive: Symptom‐triggered electrocardiograms performed as needed.
Multicenter CRRF‐PeAF [12]	Japan	499	Arctic Front Advance, Medtronic (Second‐Fourth Generation)	Contact‐force‐sensing irrigated ablation catheter	12	Scheduled: Twice‐daily one‐channel ambulatory ECGs for the entire 12‐month follow‐up, supplemented by 24‐hour Holter monitoring at 3 and 12 months, and clinical 12‐lead ECG evaluations at 1 month and every 1–3 months thereafter.
						Adjunctive: Symptom‐triggered ambulatory ECG recordings performed as needed throughout the 1‐year follow‐up period.
Multicenter EXCAPE‐AF [13]	South Korea	288*	Arctic Front Advance, Medtronic (Second‐Fourth Generation)	Contact‐force‐sensing irrigated ablation catheter	12	Scheduled: Clinical assessments and 12‐lead ECGs at baseline, 3, 6, and 12 months; 24‐hour Holter monitoring at 3, 6, and 12 months; and 2‐week patch ECG monitoring at 3‐ and 12‐month post‐randomization.
						Adjunctive: Additional outpatient visits (with 12‐lead ECGs) scheduled as needed for routine care.

### 3.2 Quality Assessment

Risk of bias assessment using the Cochrane RoB‐2 tool revealed acceptable methodological quality overall (Figure 2). All five included trials were assigned an overall judgment of “some concerns”. This classification was primarily driven by the inherently open‐label nature of catheter ablation trials, which precludes blinding of patients and operating electrophysiologists, thereby introducing minor methodological concerns regarding deviations from intended interventions. Notably, while most trials carried some concerns for outcome measurement bias due to their reliance on intermittent Holter or patch monitoring in an unblinded setting, Baimbetov et al. was judged to be at low risk of bias in this domain [10]. This exception was due to their utilization of continuous automated rhythm surveillance via implantable cardiac monitors, which ensured highly objective, device‐adjudicated endpoint ascertainment independent of investigator influence. Furthermore, all trials demonstrated a low risk of bias concerning missing outcome data, maintained exceptionally high follow‐up completion rates, and employed robust, prespecified randomization protocols. These fundamental methodological strengths largely mitigate the potential for clinically significant detection or attrition bias, supporting the overall validity and robustness of the pooled estimates.

**FIGURE 2:**
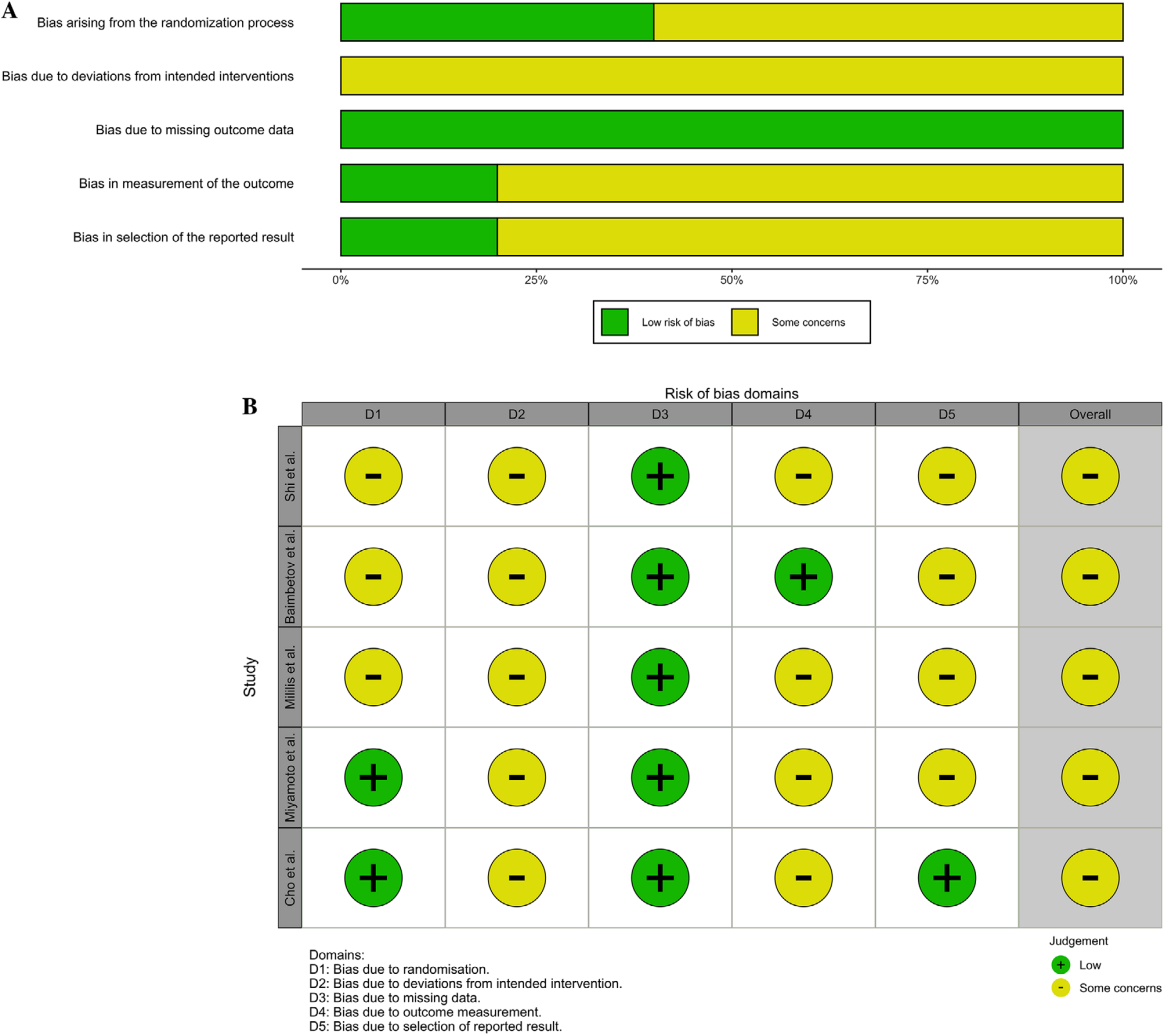
Risk of bias assessment of included randomized clinical trials. Assessment was performed using the Cochrane Risk of Bias 2 (RoB‐2) tool. (A) Weighted summary plot illustrating the proportion of studies with low risk or some concerns for each domain; notably, no studies were judged to be at high risk of bias. (B) Study‐level “traffic light” plot detailing judgments for each domain across the 5 included trials. Overall risk of bias was judged as having “some concerns” for all included studies, primarily driven by the inherently non‐blinded nature of the interventions. D1, Randomization process; D2, Deviations from intended interventions; D3, Missing outcome data; D4, Measurement of the outcome; D5, Selection of the reported result. [Colour figure can be viewed at wileyonlinelibrary.com]

### 3.3 Efficacy Outcomes

#### 3.3.1 Any Arrhythmia Recurrence at 12 Months

Pooled analysis demonstrated no significant difference in the composite endpoint of any atrial arrhythmia recurrence at 12 months between CBA and RFA (RR: 0.97; 95% CI: 0.81–1.15; *I* ^2^ = 0%; *p* = 0.71) (Figure 3). Unadjusted event rates were 29.9% and 30.2% in the CBA and RFA arms, respectively.

**FIGURE 3:**
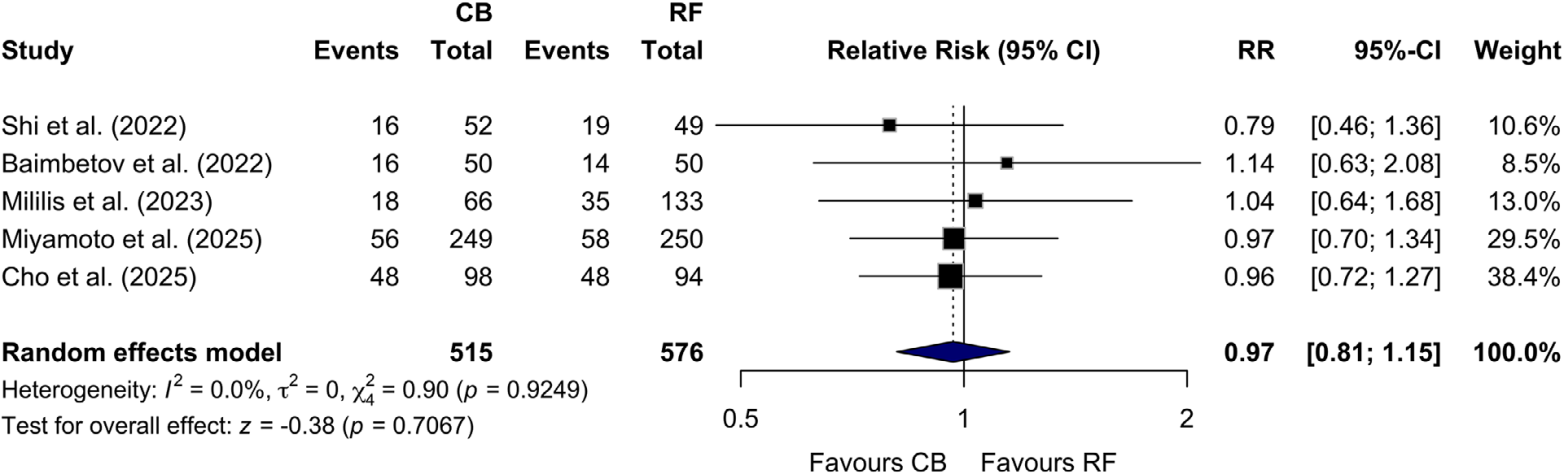
Forest plot of atrial arrhythmia recurrence at 12 months. Pooled analysis of 5 RCTs demonstrating no statistically significant difference in rhythm outcomes between cryoballoon and RFA strategies. CB, cryoballoon; CI, confidence interval; RF, radiofrequency; RR, relative risk. [Colour figure can be viewed at wileyonlinelibrary.com]

#### 3.3.2 Atrial Fibrillation Recurrence at 12 Months

Isolated AF recurrence at 12 months was comparable between groups (RR: 0.90; 95% CI: 0.63–1.29; *I* ^2^ = 23.5%; *p* = 0.57) (Figure 4), with unadjusted rates of 21.6% (CBA) vs. 23.1% (RFA). The confidence interval broadly encompasses the null, confirming no statistically significant difference.

**FIGURE 4:**
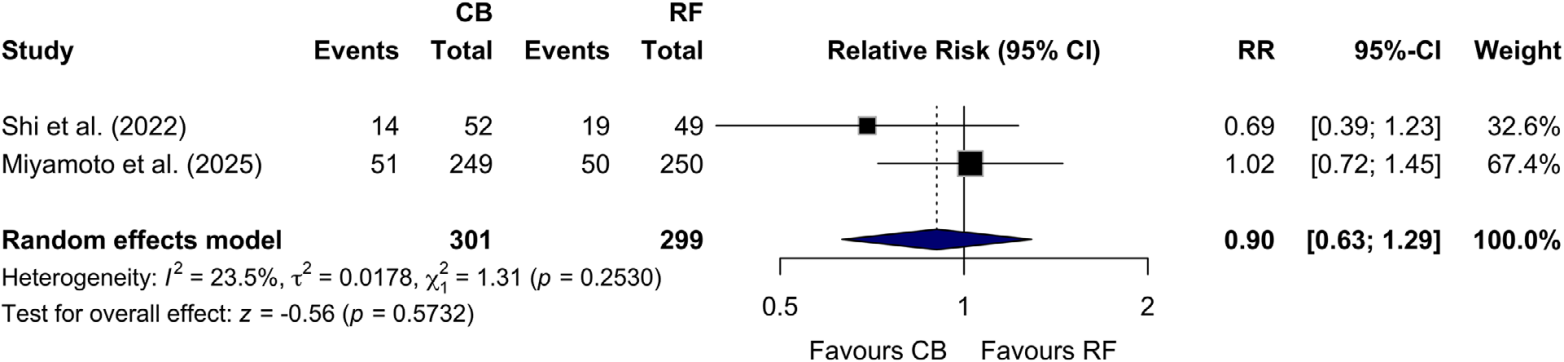
Forest plot of atrial fibrillation recurrence at 12 months. Pooled analysis of 2 RCTs comparing cryoballoon and RFA strategies. CB, cryoballoon; CI, confidence interval; RF, radiofrequency; RR, relative risk. [Colour figure can be viewed at wileyonlinelibrary.com]

#### 3.3.3 Atrial Tachycardia and Atrial Flutter Recurrence at 12 Months

Among 600 patients with analyzable data, AT/AFL recurrence rates at 12 months did not differ significantly between groups (RR: 0.40; 95% CI: 0.11–1.40; *I* ^2^ = 30.6%; *p* = 0.15) (Figure 5), with numerically lower unadjusted rates in the CBA arm (1.7% vs. 4.3%).

**FIGURE 5:**
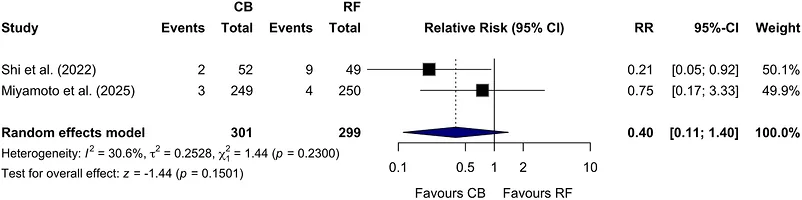
Forest plot of atrial tachycardia and atrial flutter recurrence at 12 months. Pooled analysis of 2 RCTs demonstrating no statistically significant difference in atrial tachycardia and atrial flutter recurrence at 12 months between cryoballoon and RFA strategies. CB, cryoballoon; CI, confidence interval; RF, radiofrequency; RR, relative risk. [Colour figure can be viewed at wileyonlinelibrary.com]

#### 3.3.4 Time‐to‐Event Analysis

In the pooled time‐to‐event analysis, CBA was associated with a comparable rate of atrial arrhythmia recurrence over 12 months relative to RFA (HR: 0.91; 95% CI: 0.67–1.24; *p* = 0.56; *I* ^2^ = 0%) (Figure 6), with no evidence of between‐study heterogeneity.

**FIGURE 6:**
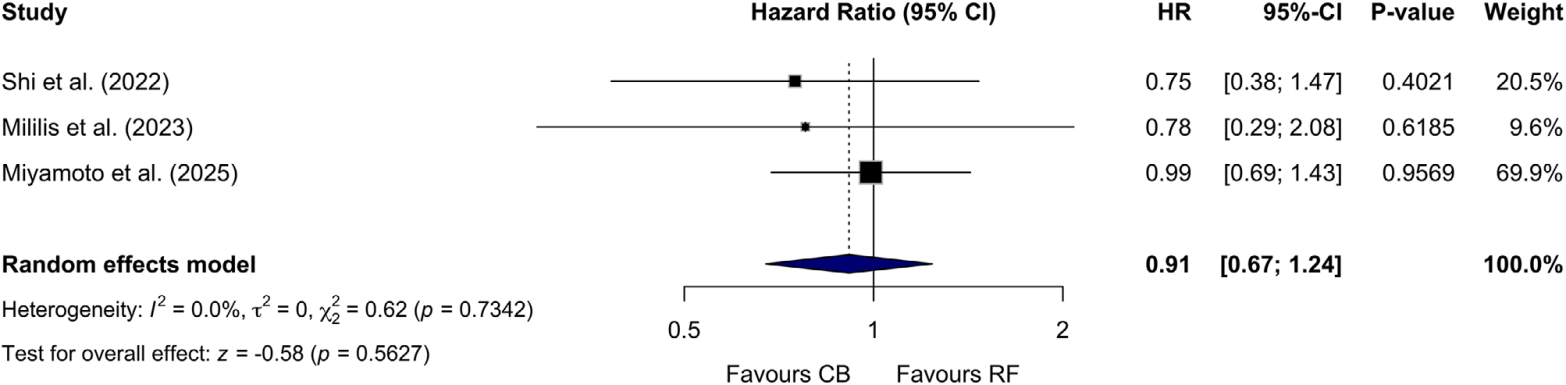
Forest plot of the time‐to‐event meta‐analysis for any atrial arrhythmia. Pooled time‐to‐event analysis of 3 RCTs demonstrating no statistically significant difference in the hazard of any atrial arrhythmia recurrence at 12 months between cryoballoon and RFA strategies. CB, cryoballoon; CI, confidence interval; HR, hazard ratio; RF, radiofrequency. [Colour figure can be viewed at wileyonlinelibrary.com]

#### 3.3.5 Trial Sequential Analysis

TSA for any arrhythmia recurrence at 12 months showed that the accrued sample (*n* = 1,091) did not reach the calculated RIS of 1704 (*D* ^2^ = 0%) required to detect a 20% relative risk reduction (Figure 7). However, the cumulative *Z*‐score crossed the prespecified futility boundary before reaching the RIS, establishing statistical futility and indicating that further enrollment is unlikely to demonstrate superiority for either modality.

**FIGURE 7:**
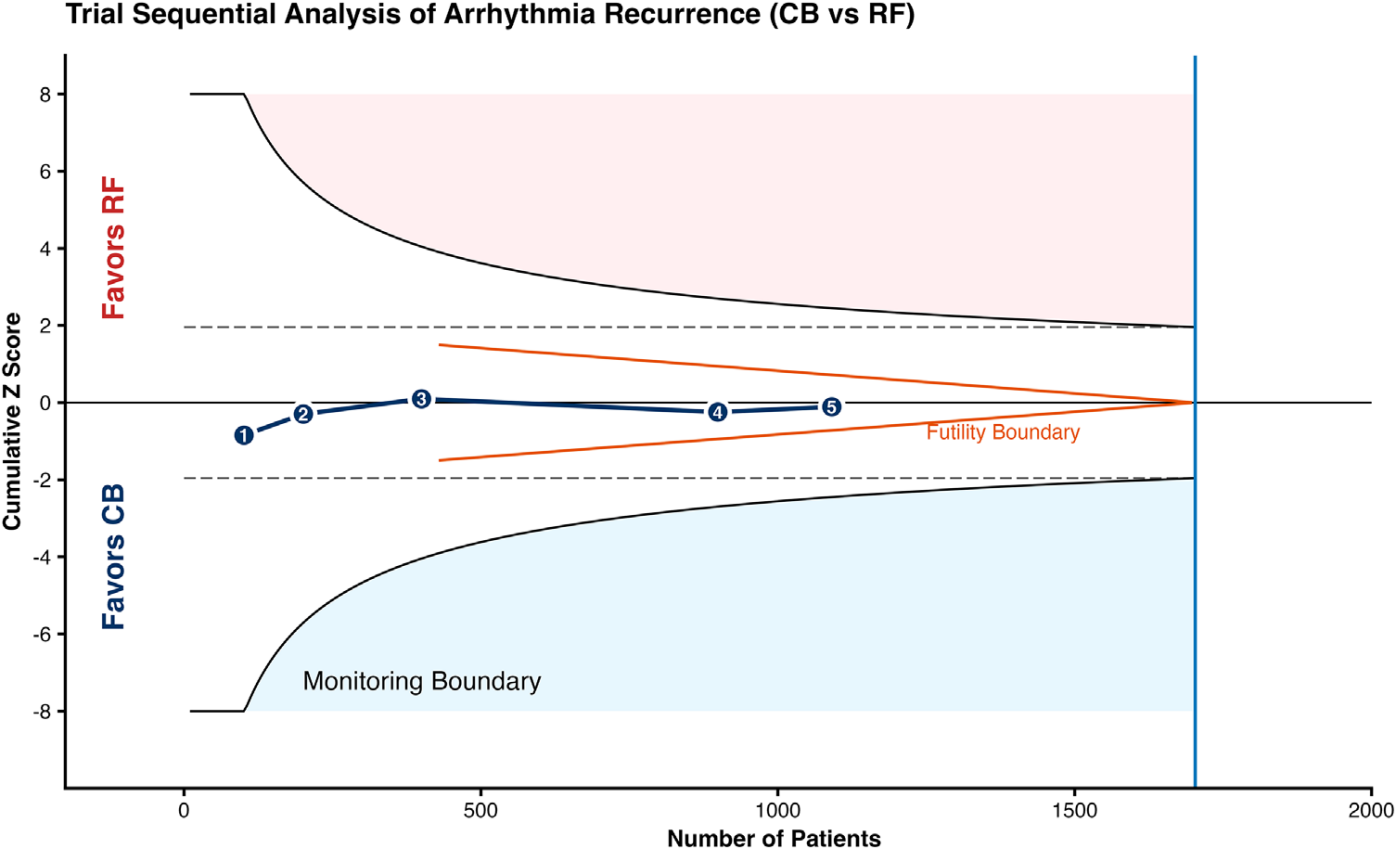
Trial sequential analysis of arrhythmia recurrence trial sequential analysis for any arrhythmia recurrence at 12 months. The blue numbered line indicates cumulative *Z*‐scores; curved lines indicate monitoring boundaries; and red wedges indicate futility boundaries. The vertical blue line represents the required information size. Numbered data points represent the cumulative addition of studies in chronological order: 1, Shi et al; 2, Baimbetov et al; 3, Mililis et al; 4, Miyamoto et al; and 5, Cho et al. Parameters: Two‐sided α = 5%, Power = 80%, Relative Risk Reduction = 20%. CB, cryoballoon; RF, radiofrequency. [Colour figure can be viewed at wileyonlinelibrary.com]

#### 3.3.6 Procedural Efficiency

The CB strategy was associated with a significantly shorter total procedural time compared to RFA (MD −45.37 min; 95% CI −61.73 to −29.01; *I* ^2^ = 90.2%, *p* < 0.0001) (Figure 8A). However, substantial heterogeneity was observed for this metric. Fluoroscopy exposure time did not differ significantly between groups, despite a numerical increase in the CB cohort, and demonstrated substantial heterogeneity (MD +3.60 min; 95% CI −4.94 to 12.14; *I* ^2^ = 95.6%, *p* = 0.41) (Figure 8B).

**FIGURE 8:**
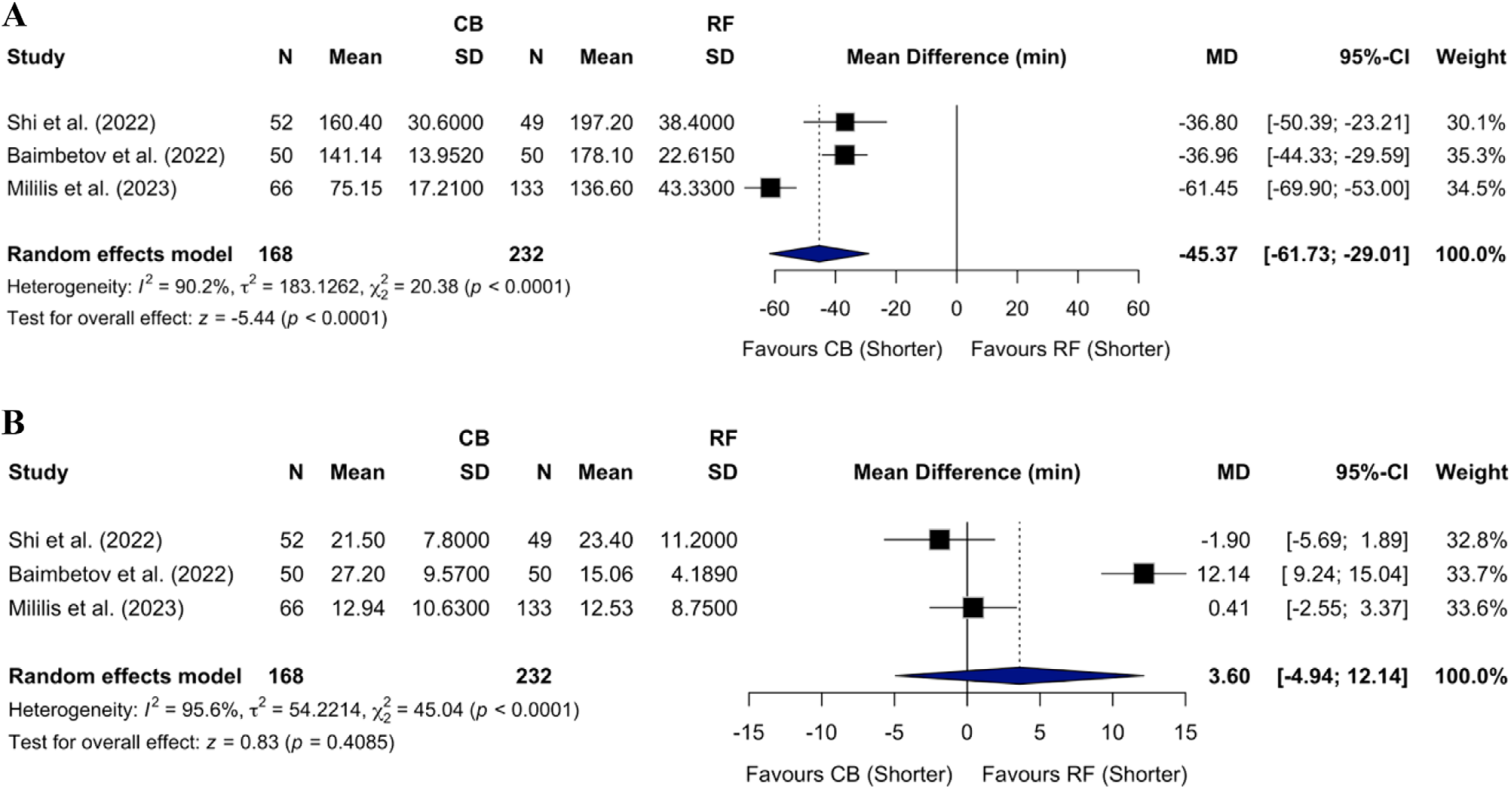
Forest plot of procedural efficiency metrics. Forest plots illustrating pooled mean differences for (A) total procedural time and (B) fluoroscopy time. CB, cryoballoon; CI, confidence interval; MD, mean difference; RF, radiofrequency; SD, standard deviation. [Colour figure can be viewed at wileyonlinelibrary.com]

#### 3.3.7 Repeat Ablation

The CB strategy did not confer a significant reduction in the need for subsequent ablation procedures. The unadjusted repeat ablation rate was 31.0% (113/365) in the CB group compared with 31.0% (134/433) in the RF group, yielding a relative risk of 0.93 (95% CI 0.76–1.14; *I* ^2^ = 0.0%, *p* = 0.50) (Figure 9). Heterogeneity was minimal, indicating a consistently comparable repeat procedure burden between the two ablation modalities.

**FIGURE 9:**
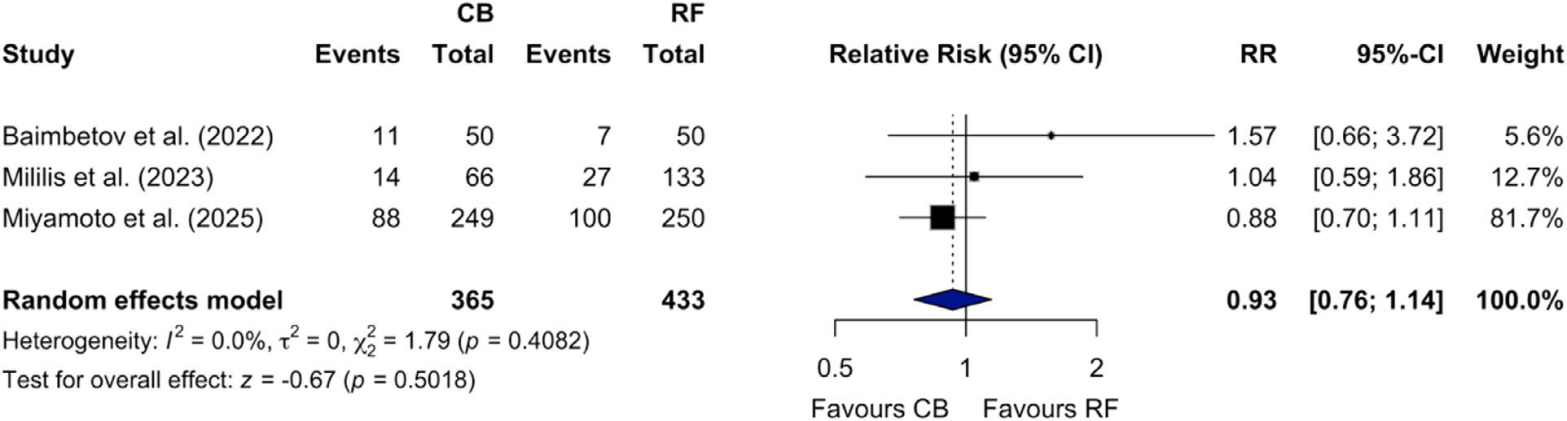
Forest plot of repeat ablation. Pooled analysis of 3 RCTs demonstrating no statistically significant difference in repeat ablation rates between cryoballoon and RFA strategies. CB, cryoballoon; CI, confidence interval; RF, radiofrequency; RR, relative risk. [Colour figure can be viewed at wileyonlinelibrary.com]

#### 3.3.8 Safety and Procedural Complications

Both ablation strategies demonstrated an excellent safety profile, yielding comparable overall complication rates. The unadjusted incidence of any procedural complication was 1.51% (7 of 465 patients) in the CB group versus 2.66% (14 of 526 patients) in the RF group. Pooled analysis confirmed no statistically significant difference in the relative risk of overall complications between the two modalities (RR: 0.55; 95% CI: 0.23–1.32; *Z* = −1.35, *p* = 0.18) (Figure 10). Heterogeneity for this endpoint was absent (*I* ^2^ = 0.0%, *p* = 0.94). Regarding specific adverse events, phrenic nerve palsy occurred exclusively in the CB cohort (0.72% vs. 0.00%), whereas access site complications (0.24% vs. 1.04%) and pericardial effusions (0.22% vs. 0.76%) were numerically less frequent in the CB arm. However, absolute event rates for all individual complications remained exceptionally low across both study cohorts.

**FIGURE 10:**
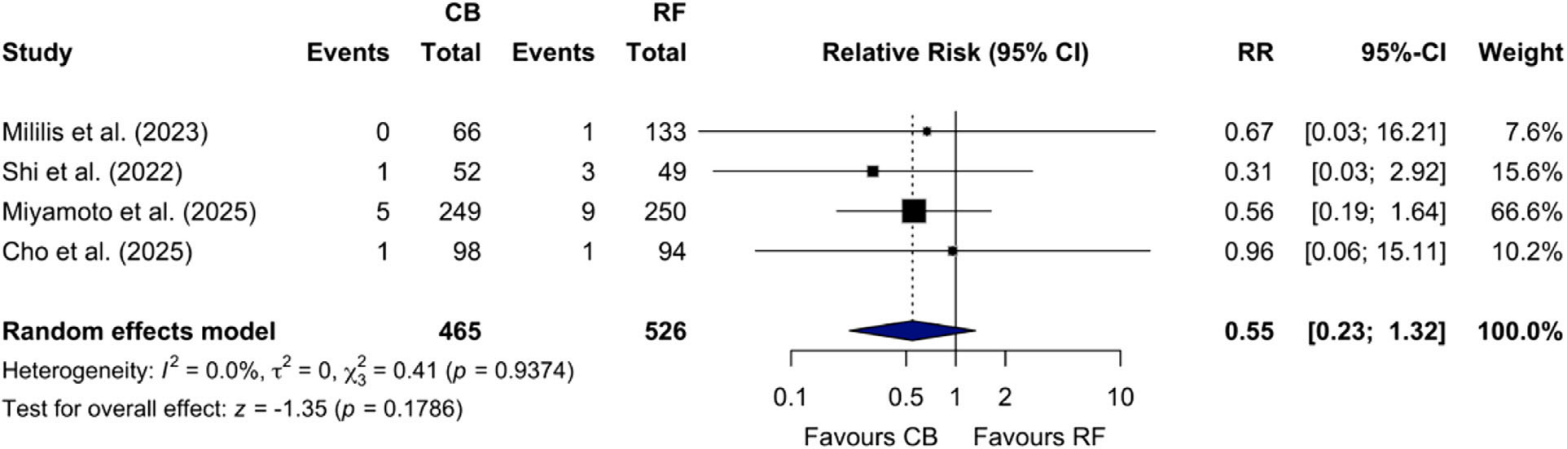
Forest plot of overall procedural complications. Pooled analysis of 4 RCTs demonstrating no statistically significant difference in the risk of overall procedural complications between cryoballoon and radiofrequency ablation strategies. CB, cryoballoon; CI, confidence interval; RF, radiofrequency; RR, relative risk. [Colour figure can be viewed at wileyonlinelibrary.com]

#### 3.3.9 Sensitivity Analyses

Sensitivity analyses confirmed the robustness of the majority of the pooled findings (see Figures S1–S7). For the primary composite endpoint of any arrhythmia recurrence, as well as isolated AF recurrence, total procedural time, fluoroscopy time, repeat ablation burden, and overall complications, sequential omission of individual studies using a leave‐one‐out approach yielded pooled estimates that remained highly stable. Across these domains, no single trial materially altered the magnitude or statistical significance of the results, demonstrating that the overall treatment effects are consistently reproducible and not driven by outlier study data. However, the analysis identified statistical fragility in the specific endpoint of AT/AFL recurrence; omission of the Miyamoto et al. trial shifted the non‐significant pooled estimate to nominal statistical significance favoring CBA (RR 0.21; 95% CI 0.05–0.92; *p* = 0.038) [12]. Consequently, while the principal efficacy, safety, and operational efficiency findings are definitively robust, comparative inferences regarding isolated AT/AFL recurrence should be interpreted with caution due to sparse event data and study‐level sensitivity. Visual inspection of the funnel plots revealed general symmetry for freedom from any arrhythmia, atrial fibrillation, and AT/AFL (Figures S8‐S10), suggesting a low likelihood of substantial publication bias; however, this assessment remains exploratory due to the small number of included trials

## 4 Discussion

This systematic review and meta‐analysis of five RCTs enrolling 1,091 patients with PeAF represents the most statistically powered head‐to‐head comparison of CBA versus RFA for PVI in this arrhythmia substrate to date. The distinct incremental value of our analysis over prior works, lies not only in expanding the rigorous, randomized data pool to five trials, but in being the first to employ a time‐to‐event meta‐analysis to dynamically evaluate long‐term rhythm durability, paired with TSA to definitively establish statistical futility and prove that further randomization is highly unlikely to alter the conclusion of clinical equivalence [14]. Both modalities demonstrated equivalent efficacy across all endpoints at 12 months. There were no statistically significant differences in composite atrial arrhythmia, isolated AF, or AT/AFL recurrence, with unadjusted recurrence rates of 29.9% for CBA and 30.2% for RFA—a clinically negligible difference. The pooled time‐to‐event analysis corroborated this equivalence, and TSA analysis confirmed definitive statistical futility, indicating that the absence of a superiority signal is robust and that further randomization is unlikely to alter this conclusion. Consistent with the equivalence in primary efficacy outcomes, the requirement for repeat ablation after the index procedure was statistically identical between the two modalities. The principal domain of clinically meaningful differentiation between the two approaches was procedural efficiency, with CBA demonstrating a highly significant reduction in total procedural time (*p* < 0.0001), an advantage that carries direct operational and patient‐level relevance in high‐volume ablation programs, independent of the equivalent rhythm outcomes established by this analysis.

### 4.1 Cryoballoon Efficacy Within the Persistent AF Substrate

The approximate 30% arrhythmia recurrence observed at 12 months across both modalities corroborates the reported recurrence rates of 33%–57% for PeAF populations, likely reflecting the fundamental biological constraints of PVI‐only strategies rather than any intrinsic limitation of cryothermal energy delivery [19, 20]. This biological ceiling is further supported by controlled trials such as STAR‐AF II, which demonstrated no incremental benefit from complex fractionated electrogram or linear lesion ablation beyond PVI, and by CAPLA which similarly showed no significant improvement in arrhythmia‐free survival with posterior wall isolation added to PVI‐only ablation in PeAF [2, 21]. In this context, the present meta‐analysis, consistently confirms that, within a PVI‐only approach, the choice of thermal energy modality does not determine rhythm outcome in PeAF. The EXCAPE‐AF trial, which randomized patients with PeAF to extended CBA (eCBA; PVI plus posterior wall isolation), standard CBA, or RFA, provides a directly relevant extension of this paradigm [13]. In this present analysis, only the sCBA arm retained against the RFA comparator to preserve PVI‐only procedural homogeneity and avoid unit‐of‐analysis errors. EXCAPE‐AF demonstrated that eCBA with adjunctive posterior wall isolation conferred no significant improvement in arrhythmia‐free survival over sCBA at 12 months, with comparable recurrence rates and no statistically significant differences across the three arms (*p* = 0.787) [13]. However, the CRRF‐PeAF trial permitted adjunctive ablations at the operator's discretion. This resulted in a significant and asymmetric application of extended lesion sets between their treatment groups, with RFA patients receiving significantly more posterior wall isolation (15.6% vs. 8.0%), superior vena cava isolation (10.4% vs. 4.8%), and cavotricuspid isthmus ablation (22.8% vs. 13.3%), while CBA patients received significantly more roof lines^12^. To ensure this discretionary substrate modification did not invalidate our primary findings, we conducted a leave‐one‐out analysis for freedom from any arrhythmia (Figure S1). Omitting the CRRF‐PeAF trial yielded a relative risk of 0.97 (95% CI: 0.78–1.19, *I* ^2^ = 0%), perfectly aligning with our overall pooled estimate. This mathematically confirms that while discretionary extended lesions introduced procedural variance, they did not disproportionately skew the core efficacy conclusions comparing CBA and RFA. This finding is mechanistically concordant with the STAR‐AF II study and CAPLA datasets, collectively indicating that supplemental substrate modification, whether delivered by RF or cryothermal energy, does not translate into incremental efficacy over PVI alone in unselected PeAF populations at 1‐year follow‐up, likely because the underlying atrial myopathy is not addressable by anatomically defined lesion extension per se [2, 21]. Taken together, these data place a ceiling on the conceptual ‘versatility’ argument for CB‐based extended ablation in PeAF: although the platform is technically adaptable to posterior wall and non‐PV ablation, the current controlled evidence does not support a superior rhythm outcome profile compared with PVI‐only strategies. Future studies evaluating single‐shot technologies in PeAF may need to look beyond anatomically defined atrial lesions toward adjunctive chemical substrate modification such as ethanol infusion into the vein of Marshall, to overcome this biological ceiling [22, 23].

Beyond primary rhythm outcomes, it is important to acknowledge the secondary structural findings reported in the CRRF‐PeAF trial. Miyamoto et al. observed significantly greater left atrial reverse modeling following RFA compared to CBA (median left atrial volume index [LAVI] change: −11 mL/m^2^ [IQR, −19 to −4] vs. −4 mL/m^2^ [IQR, −13 to 3], *p* < 0.001) [12]. Left atrial reverse modeling, characterized by a reduction in atrial volume and the recovery of mechanical function following the restoration of sinus rhythm, is recognized as an important surrogate marker for the attenuation of atrial myopathy and a strong predictor of long‐term freedom from AF recurrence [24, 25]. However, the enhanced volumetric reduction seen with RFA in the CRRF‐PeAF trial may be mechanistically linked to the more extensive, discretionary substrate modification (such as posterior wall isolation) that was performed significantly more frequently in that cohort [12, 26]. Despite the known correlation between LAVI reduction and arrhythmia freedom, our pooled analysis demonstrates equivalent 12‐month rhythm outcomes between the two thermal modalities [27]. This suggests that while RFA may induce a more pronounced early volumetric reduction in workflows utilizing extensive adjunctive ablation sets, this structural difference does not translate into a definitive clinical advantage at one year. It remains plausible that the true antiarrhythmic benefits of enhanced left atrial reverse remodeling manifest only over a more extended time horizon [24]. Consequently, longitudinal studies with follow‐up extending beyond 12 months are required to determine whether the superior structural reverse remodeling associated with RFA ultimately confers a long‐term clinical benefit in protecting against late PeAF recurrence. Furthermore, future trial designs must pre‐specify adjunctive lesion strategies across both arms to mitigate the confounding variable observed in the CRRF‐PeAF trial.

### 4.2 Procedural Efficiency: The Clinically Distinguishing Advantage of CBA

While efficacy is equivalent, procedural efficiency represents the most clinically meaningful differentiator in the present analysis. CBA was associated with a highly significant reduction in total procedural time (MD −45.37 min; 95% CI: −67.73 to −29.01; *p* < 0.0001), consistent in direction across all five constituent trials and corroborating prior meta‐analytic evidence demonstrating that CBA shortens total procedure time by approximately 30–45 min compared with RFA [28]. However, the substantial procedure‐time heterogeneity (*I* ^2^ = 90.2%) reflects center‐specific differences in workflow, operator experience, and institutional protocols, rather than marked variation in catheter or mapping systems, as the included trials predominantly used second‐ to fourth‐generation Arctic Front Advance cryoballoons (Medtronic) and contact‐force‐sensing irrigated RFA catheters with relatively similar mapping platforms. For procedural time, leave‐one‐sensitivity analysis (Figure S4) identified Mililis et al. as the sole driver of heterogeneity; omitting this study reduced the I^2^ statistic from 90.2% to 0% (yielding a revised mean difference of −36.92 min favoring CBA) [11]. Clinically, this is explained by highly divergent ablation protocols: Mililis et al. employed a single 240‐second freeze per vein, intrinsically expediting the procedure, whereas Shi et al. mandated two 240‐second freezes for every vein regardless of initial isolation [9, 11]. Furthermore, Baimbetov et al. enforced a mandatory 30 min observation period post‐ablation to strictly assess for early pulmonary vein reconnection, naturally inflating total procedural duration across their cohort [10].

This efficiency is mechanistically attributable to the single‐shot design, which eliminates repetitive catheter repositioning, enforces fixed standardized lesion durations per vein, and reduces reliance on electroanatomical mapping systems. While time‐to‐effect dosing strategies have been shown to further reduce CBA procedural times in paroxysmal cohorts without compromising acute success or short‐term outcomes, direct evidence in PeAF remains limited [29]. Therefore, the present meta‐analysis provides the most robust head‐to‐head assessment of baseline CB‐mediated time savings within a PeAF‐specific framework. Fluoroscopy time did not differ significantly between modalities (*p* = 0.41), consistent with prior comparative data showing that CBA generally reduces fluoroscopy exposure relative to mapping‐heavy RF strategies, and contemporary fluoroscopy‐reduction or even fluoroless techniques such as intracardiac echocardiography, are progressively attenuating any residual radiation differential [30, 31, 32]. Similarly, our leave‐one‐out sensitivity analysis for fluoroscopy time (Figure S5) identified Baimbetov et al. as the singular driver of variance; its omission dropped the *I* ^2^ from 95.6% to 0% [10]. This profound variability is highly sensitive to institutional mapping practices. RFA procedures in the contemporary era increasingly utilize zero‐fluoroscopy 3D electroanatomical mapping systems [33]. In contrast, standard CBA protocols, particularly the approach utilized in Baimbetov et al., which relied on traditional fluoroscopic control and routine venography without the mitigating benefit of 3D mapping integration, necessitated significantly higher radiation exposure, skewing the pooled estimates [10].

Importantly, the procedural‐time advantage of CBA carries no down‐stream cost in terms of repeat ablation burden: the requirement for repeat ablation following the index procedure was statistically identical between groups (31.0% vs. 31.0%), with near‐zero heterogeneity confirming consistency across the trial pool. In high‐volume ablation programs, a 45 min per‐case time reduction translates into meaningful gains in laboratory capacity, and improved procedural capacity, as demonstrated in recent efficiency‐modeling studies. Specifically, a lab efficiency sub‐analysis of the Cryo Global Registry used discrete‐event simulation to show that three CB‐based PVI procedures could be performed in a single lab day in most simulated scenarios, with more than two‐thirds of days allowing for an additional simple EP procedure (e.g., pacemaker implant or device replacement) and staff overtime required in less than 15% of days [34, 35]; adoption of time‐saving techniques such as the ‘figure‐of‐eight’ closure further reduced overtime and increased the proportion of days with capacity for an extra procedure [36]. These operational benefits accrue at scale independently of the equivalent rhythm outcomes established by this analysis and reinforce the role of CBA as a procedural‐efficiency‐driven strategy within the paradigm for PeAF, particularly in resource‐constrained or high‐demand healthcare systems.

### 4.3 Safety Profile and Phrenic Nerve Palsy

Both ablation strategies demonstrated comparable procedural safety in the present analysis. The unadjusted incidence of any procedure‐related complication was 1.51% in the CBA group versus 2.66% in the RFA group. In the present cohort, access‐site complications (0.24% vs. 1.04%; *p* = 0.17) and pericardial effusions (0.22% vs. 0.76%; *p* = 0.43) were numerically lower in the CBA arm, consistent with the broader safety profile of single‐shot CB‐based PVI [37, 38]. PNP occurred exclusively in the CBA cohort (0.72% vs. 0.00%; *p* = 0.25), an anatomically predictable asymmetry arising from the proximity of the right phrenic nerve to the right superior and inferior pulmonary vein ostia during right‐sided freeze lesions. Given the low absolute event counts across all individual complication endpoints in the present analysis, these differences did not achieve statistical significance and safety inferences should be drawn with corresponding circumspection. This observation is nonetheless consistent with contemporary data: in the FIRE‐AND‐ICE trial, in which PNP rates were 2.7% with CBA versus 0% with RFA (p < 0.01), and the worldwide YETI registry reports that approximately 4.2% of patients undergoing CB‐based PVI experience phrenic nerve injury, with 97% recovering within 12 months and permanent symptomatic PNP occurring in fewer than 0.1% of cases [7, 39, 40]. Intraprocedural monitoring of right phrenic nerve function is the established mitigation strategy for PNP during CB‐based right‐sided PV ablation [41]. This approach relies on immediate cryoenergy termination upon a ≥30% reduction in amplitude from baseline and is formally endorsed by the EHRA/HRS/APHRS/LAHRS expert consensus statement [1].

### 4.4 Limitations

Several methodological and follow‐up‐related limitations warrant acknowledgement. First, the evidence base is predominantly anchored at 12 months, as four of the five included trials reported efficacy outcomes at 1 year post‐ablation. Although one trial (Baimbetov et al.) extended follow‐up to 36 months, this single‐center cohort lacks the statistical weight to drive long‐term pooled estimates [10]. Given that PVI‐only strategies in the PeAF substrate are characterized by a progressive, time‐dependent rise in atrial arrhythmia recurrence, this 12‐month synthesis likely underestimates late arrhythmia recurrence and may overestimate the true long‐term durability of these ablation modalities [42]. A second substantive limitation is heterogeneity in arrhythmia‐monitoring methodology across the five included trials, which directly confounds the comparability of pooled recurrence estimates. Surveillance strategies ranged markedly in intensity (see Table 1). Most protocols relied on intermittent clinic visits accompanied by short‐term Holter or patch‐based ambulatory ECG monitoring, which are known to under‐detect asymptomatic and brief AF episodes when compared with continuous device‐based surveillance [8, 43, 44]. In contrast, the Baimbetov et al. trial employed implantable electrocardiographic loop recorders with continuous automated rhythm surveillance and still reported comparable freedom from recurrent arrhythmia between modalities (Kaplan–Meier estimates of 68% for CBA and 72% for RFA at 3 years), confirming the absence of a clinically significant difference even under intensive monitoring [10]. Furthermore, 12‐month recurrence data for isolated AF and AT/AFL were formally reported in only two trials, resulting in sparse reporting for the secondary efficacy variables. This sparsity reduces the precision of the pooled effect estimates for arrhythmia‐specific outcomes and increases the risk of random‐error‐driven inference.

### 4.5 Future Directions

Several critical research gaps warrant investigation to fully optimize the ablation paradigm for the PeAF substrate. First, randomized comparative effectiveness trials directly contrasting CBA against emerging single‐shot alternatives like PFA are currently lacking in PeAF [45]. While the present meta‐analysis establishes the clinical equivalence of CBA and RFA in PeAF, these thermal modalities must now be contextualized within the rapidly evolving landscape of PFA. Utilizing high‐voltage electrical pulses to induce irreversible electroporation, PFA offers a non‐thermal alternative characterized by functional cardiac tissue selectivity. This fundamental biophysical advantage minimizes collateral injury to critical periatrial structures, such as the esophagus and phrenic nerve, positioning PFA as a formidable comparator for future arrhythmia management paradigms [46, 47]. Recent comparative data suggest that PFA yields at least comparable, and potentially superior outcomes relative to established thermal modalities across AF subtypes [48]. In patients with paroxysmal AF, the PERFECT‐PAF trial demonstrated an 86.6% freedom from atrial tachyarrhythmia at 12 months for PFA compared to 77.8% for CBA [49]. Comparably, a recent Bayesian meta‐analysis encompassing 3120 patients demonstrated that PFA was associated with a higher freedom from atrial tachyarrhythmia recurrence compared to both RFA and CBA (HR 0.68; 95% credible interval 0.55–0.84) [50]. Evidence supporting PFA within the specific substrate of PeAF is also maturing. In a recent cohort study evaluating a PVI‐only approach for PeAF, Isenegger et al. reported a 12‐month recurrence free survival of 72% for PFA versus 60% for CBA [45]. Notably, patients treated with PFA exhibited significantly higher frequency of regression from persistent to paroxysmal AF (68% vs. 37%), suggesting potentially superior modification of arrhythmogenic substrate. These efficacy signals are further corroborated by large prospective studies, such as the ADVANTAGE AF trial which reinforces the durability of PFA in persistent cohorts [51]. However, despite these promising early signals, the current body of evidence for PFA remains predominantly anchored in paroxysmal AF populations. Data regarding the efficacy of PFA in PeAF, particularly concerning long‐term outcomes and reconnection rates beyond one year of follow‐up remain limited. While recent observational registries and prospective cohorts report promising PFA efficacy, head‐to‐head trials, notably the anticipated PEACE study (NCT07064616), are required to clarify whether these non‐randomized signals translate into superior efficacy‐safety trade‐offs, or whether the persistent substrate imposes a universal ceiling across all single‐shot modalities [50, 51, 52, 53].

Second, although CBA has been established as a highly cost‐effective strategy relative to antiarrhythmic drug therapy in paroxysmal AF, analogous data for PeAF cohorts remain conspicuously absent [54]. Furthermore, dedicated health economic analyses incorporating procedural time savings, repeat ablation burden, and quality‐adjusted life years are required to contextualize the clinical equivalence established in this analysis within broader health system frameworks.

## 5 Conclusion

In patients with PeAF, CBA, and RFA achieved similar 12‐month rhythm outcomes and repeat‐ablation rates, indicating that thermal source selection does not materially change arrhythmia suppression in this substrate. However, the consistent reduction in procedural time with CBA suggests that its principal advantage lies in procedural efficiency and workflow implication rather than superior efficacy. Accordingly, CBA should be viewed as a noninferior rhythm‐control strategy with an operational benefit, while future studies should focus on longer‐term follow‐up, standardized rhythm surveillance, and direct comparisons with emerging non‐thermal technologies.

## Funding

This research did not receive any specific grant from funding agencies in the public, commercial, or not‐for‐profit sectors.

## Ethics Statement

Ethical approval and informed consent were not required for this study, as it is a systematic review and meta‐analysis of previously published literature. All data analyzed were extracted from publicly available studies, and no human or animal subjects were directly involved or identified in this research.

## Conflicts of Interest

The authors declare no conflicts of interest.

## Supporting information




**Supporting Information**: pace70350‐Sup‐0001‐SuppMatt.pdf

## Data Availability

The data underlying this article are available in the article and in its online supplementary material.
