# Supplementary material for: Cryoballoon Versus Radiofrequency Ablation for Persistent Atrial Fibrillation: Meta‐Analysis of Randomized Trials
Source: Pacing Clin Electrophysiol. 2026 Jul 3;49(11):1692–705. doi: 10.1111/pace.70350 (PMC13739753; doi:10.1111/pace.70350)

Supplementary material

Supplementary Table S1: Search Strategy for PubMed, Embase, and Scopus

Search Terms
**PubMed Search Strategy:**<br>("Atrial Fibrillation"[Mesh] OR "atrial fibrillation"[tiab] OR "persistent atrial fibrillation"[tiab] OR "AF"[tiab] OR ("atrial"[tiab] AND "fibrillation"[tiab])) AND (("Catheter Ablation"[Mesh] OR "Ablation Techniques"[Mesh] OR "Radiofrequency Ablation"[Mesh] OR "radiofrequency ablation"[tiab] OR "radio-frequency ablation"[tiab] OR "RF ablation"[tiab] OR RFA[tiab] OR ("radiofrequency"[tiab] AND "ablation"[tiab]) OR ("radio-frequency"[tiab] AND "ablation"[tiab])) OR ("Cryosurgery"[Mesh] OR "Cryoablation"[tiab] OR "cryoballoon ablation"[tiab] OR "cryoballoon"[tiab] OR CBA[tiab] OR "cryothermal ablation"[tiab] OR ("cryo"[tiab] AND "ablation"[tiab]) OR ("cryo"[tiab] AND "balloon"[tiab]) OR ("cryogenic"[tiab] AND "ablation"[tiab]))) AND ("Randomized Controlled Trial"[Publication Type] OR "Controlled Clinical Trial"[Publication Type] OR "randomized"[tiab] OR "randomised"[tiab] OR "randomly"[tiab] OR "random allocation"[tiab] OR "random*"[tiab] OR "RCT"[tiab] OR "clinical trial"[tiab] OR "trial"[tiab]) NOT ("animals"[MeSH Terms] NOT "humans"[MeSH Terms])<br><br>**Embase:**<br>((exp Atrial Fibrillation/ OR (atrial fibrillation or persistent atrial fibrillation or AF).ti,ab. OR (atrial adj3 fibrillation).ti,ab.) AND ((exp Catheter Ablation/ OR exp Radiofrequency Ablation/ OR (radiofrequency ablation or radio-frequency ablation or RF ablation or RFA).ti,ab. OR ((radiofrequency or radio-frequency) adj3 ablation).ti,ab.) OR (exp Cryosurgery/ OR (cryosurgery or cryoablation or cryoballoon ablation or cryoballoon or cryothermal ablation or CBA).ti,ab. OR ((cryo or cryogenic) adj3 ablation).ti,ab. OR (cryo adj3 balloon).ti,ab.))) AND (randomized controlled trial.pt. OR controlled clinical trial.pt. OR (randomized or randomised or randomly or random allocation or trial or RCT).ti,ab.) NOT (exp animals/ NOT humans.sh.)<br><br>**Scopus:**<br>TITLE-ABS-KEY(("atrial fibrillation" OR "persistent atrial fibrillation" OR AF OR ("atrial" W/3 "fibrillation"))) AND TITLE-ABS-KEY(("radiofrequency ablation" OR "radio-frequency ablation" OR "RF ablation" OR RFA OR ("radiofrequency" W/3 "ablation") OR ("radio-frequency" W/3 "ablation") OR "cryosurgery" OR "cryoablation" OR "cryoballoon ablation" OR cryoballoon OR "cryothermal ablation" OR CBA OR ("cryo" W/3 "ablation") OR ("cryogenic" W/3 "ablation") OR ("cryo" W/3 "balloon"))) AND TITLE-ABS-KEY(("randomized controlled trial" OR "randomised controlled trial" OR randomized OR randomised OR randomly OR "random allocation" OR random* OR RCT OR "clinical trial" OR trial)) AND (LIMIT-TO(DOCTYPE, "ar")) AND (LIMIT-TO(SUBJAREA, "MEDI") OR LIMIT-TO(SUBJAREA, "HEAL"))

Supplementary Table S2: Baseline Characteristics of Included Studies

Characteristic	Study	Cryoballoon	Radiofrequency
**Total Patients (N)**	Shi et al.	52	49
	Baimbetov et al.	50	50
	Mililis et al.	66	133
	Miyamoto et al.	249	250
	Cho et al.	98	94
	**Overall Total**	**515**	**576**
**Age (years, Mean ± SD)** *	Shi et al.	62.4 ± 8.4	64.0 ± 8.7
	Baimbetov et al.	61.3 ± 10.2	61.6 ± 6.5
	Mililis et al.	62.74 ± 9.09	60.22 ± 9.87
	Miyamoto et al.	69 [60-74]	69 [62-74]
	Cho et al.	58.9 ± 8.8	60.3 ± 8.9
	**Overall Mean Age**	**60.99**	**61.02**
**Female (n, %)**	Shi et al.	7 (13.5%)	14 (28.6%)
	Baimbetov et al.	19 (38%)	21 (42%)
	Mililis et al.	15 (22.7%)	22 (16.7%)
	Miyamoto et al.	67 (26.9%)	69 (27.6%)
	Cho et al.	11 (11.2%)	17 (18.1%)
	**Overall Female**	**119 (23.1%)**	**143 (24.8%)**
**BMI (kg/m², Mean ± SD)** †	Shi et al.	29.6 ± 4.7	28.8 ± 4.5
	Baimbetov et al.	NR	NR
	Mililis et al.	33.07 ± 3.11	30.07 ± 5.28
	Miyamoto et al.	24.4 [22.1-26.4]	24.3 [22.2-26.7]
	Cho et al.	26.2 ± 2.7	26.2 ± 3.4
	**Overall Mean BMI**	**29.14**	**28.57**
**Hypertension, n (%)** ‡	Shi et al.	29 (55.8%)	28 (58.0%)
	Baimbetov et al.	NR	NR
	Mililis et al.	NR	NR
	Miyamoto et al.	157 (63.1%)	161 (64.4%)

	Cho et al.	54 (55.1%)	56 (59.6%)
	**Overall Hypertension**	**240 (60.2%)**	**245 (62.3%)**
**Diabetes mellitus, n (%)** [§]	Shi et al.	1 (1.9%)	4 (8.2%)
	Baimbetov et al.	NR	NR
	Mililis et al.	9 (13.6%)	20 (15.0%)
	Miyamoto et al.	40 (16.1%)	35 (14%)
	Cho et al.	15 (15.3%)	19 (20.2%)
	**Overall Diabetes**	**65 (14.0%)**	**78 (14.8%)**
**Heart failure, n (%)** [¶]	Shi et al.	NR	NR
	Baimbetov et al.	NR	NR
	Mililis et al.	NR	NR
	Miyamoto et al.	43 (17.3%)	40 (16%)
	Cho et al.	40 (16%)	10 (10.6%)
	**Overall Heart Failure**	**83 (23.9%)**	**50 (14.5%)**
**Stroke/TIA, n (%)** [#]	Shi et al.	NR	NR
	Baimbetov et al.	NR	NR
	Mililis et al.	NR	NR
	Miyamoto et al.	13 (5.2%)	19 (7.6%)
	Cho et al.	7 (7.1%)	3 (3.2%)
	**Overall Stroke/TIA**	**20 (5.8%)**	**22 (6.4%)**
**CHA_2DS_2-VASc Score (Mean ± SD)** [**]	Shi et al.	NR	NR
	Baimbetov et al.	1 ± 0.5	0.9 ± 0.6
	Mililis et al.	1.71 ± 1.37	1.61 ± 1.31
	Miyamoto et al.	1 [1-2]	1 [1-2]
	Cho et al.	1.2 ± 1.2	1.5 ± 1.1
	**Overall Mean Score**	**1.31**	**1.45**
**LA Size (mm, Mean ± SD)** [††]	Shi et al.	46 ± 6	44 ± 7
	Baimbetov et al.	41 ± 5	39 ± 7
	Mililis et al.	43.55 ± 4.74	44.89 ± 4.79

	Miyamoto et al.	41.5 [37.3-45.0]	42.0 [38.5-45.0]
	Cho et al.	43.7 ± 5.1	44.0 ± 6.2
	**Overall Mean Diameter**	**43.6**	**43.6**
**LVEF (%, Mean ± SD)** [‡‡]	Shi et al.	56.0 ± 7.2	56.8 ± 8.1
	Baimbetov et al.	59 ± 5	54 ± 6
	Mililis et al.	55.70 ± 6.16	55.15 ± 7.10
	Miyamoto et al.	60.0 [55.0-65.4]	60.0 [53.0-64.3]
	Cho et al.	NR	NR
	**Overall Mean LVEF**	**56.77**	**55.23**
**AF Duration (months, Mean ± SD)** [§§]	Shi et al.	NR	NR
	Baimbetov et al.	19 ± 17	22 ± 15
	Mililis et al.	72.8 ± 77.9	67.6 ± 45.4
	Miyamoto et al.	4 [2-6]	3 [2-6]
	Cho et al.	7.9 [4.8-15.7]	9.4 [5.3-21.0]
	**Overall Mean Duration**	**49.6**	**55.1**
**Beta-blocker, n (%)**	Shi et al.	36 (69.2%)	30 (61.2%)
	Baimbetov et al.	33 (66%)	27 (54%)
	Mililis et al.	48 (72.7%)	90 (67.7%)
	Miyamoto et al.	118 (47.4%)	112 (44.8%)
	Cho et al.	59 (60.2%)	53 (56.4%)
	**Overall Beta-blocker**	**294 (57.0%)**	**312 (54.2%)**
**Amiodarone, n (%)**	Shi et al.	14 (26.9%)	13 (26.5%)
	Baimbetov et al.	37 (74%)	41 (82%)
	Mililis et al.	37 (56.1%)	74 (55.6%)
	Miyamoto et al.	3 (1.2%)	8 (3.2%)
	Cho et al.	28 (28.6%)	26 (27.7%)
	**Overall Amiodarone**	**119 (23.1%)**	**162 (28.1%)**

Data presented as Mean ± SD, Median [IQR], or n (%). NR = Not Reported. LA = Left Atrial. LVEF = Left Ventricular Ejection Fraction.

- [*]**Age**: Pooled calculation only includes studies reporting Mean±SD (Shi et al., Baimbetov et al., Mililis et al., Cho et al.).
- [†]**BMI**: Pooled calculation only includes studies reporting Mean±SD (Shi et al., Mililis et al., Cho et al.).
- [‡]**Hypertension**: Pooled calculation only includes studies reporting this (Shi et al., Miyamoto et al., Cho et al.).
- [§]**Diabetes**: Pooled calculation only includes studies reporting this (Shi et al., Mililis et al., Miyamoto et al., Cho et al.).
- [¶]**Heart Failure**: Pooled calculation only includes studies reporting this (Miyamoto et al., Cho et al.).
- [#]**Stroke/TIA**: Pooled calculation only includes studies reporting this (Miyamoto et al., Cho et al.).
- [**]**CHA_2DS_2-VASc**: Pooled calculation only includes studies reporting Mean±SD (Baimbetov et al., Mililis et al., Cho et al.).
- [††]**LA Diameter**: Pooled calculation only includes studies reporting Mean±SD (Shi et al., Baimbetov et al., Mililis et al., Cho et al.).
- [‡‡]**LVEF**: Pooled calculation only includes studies reporting Mean±SD (Shi et al., Baimbetov et al., Mililis et al.).
- [§§]**AF Duration**: Pooled calculation only includes studies reporting Mean±SD (Baimbetov et al., Mililis et al.).

Supplementary Figure S1: Leave-one-out sensitivity analysis for freedom from any arrhythmia.

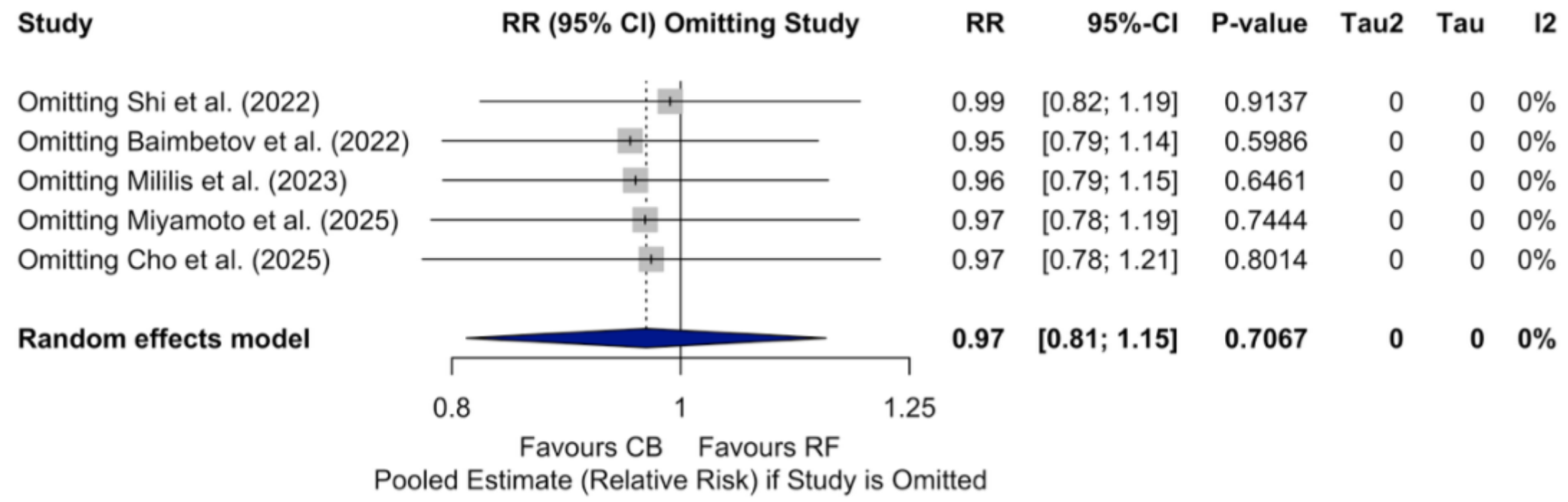

Supplementary Figure S2: Leave-one-out sensitivity analysis for freedom from AF.

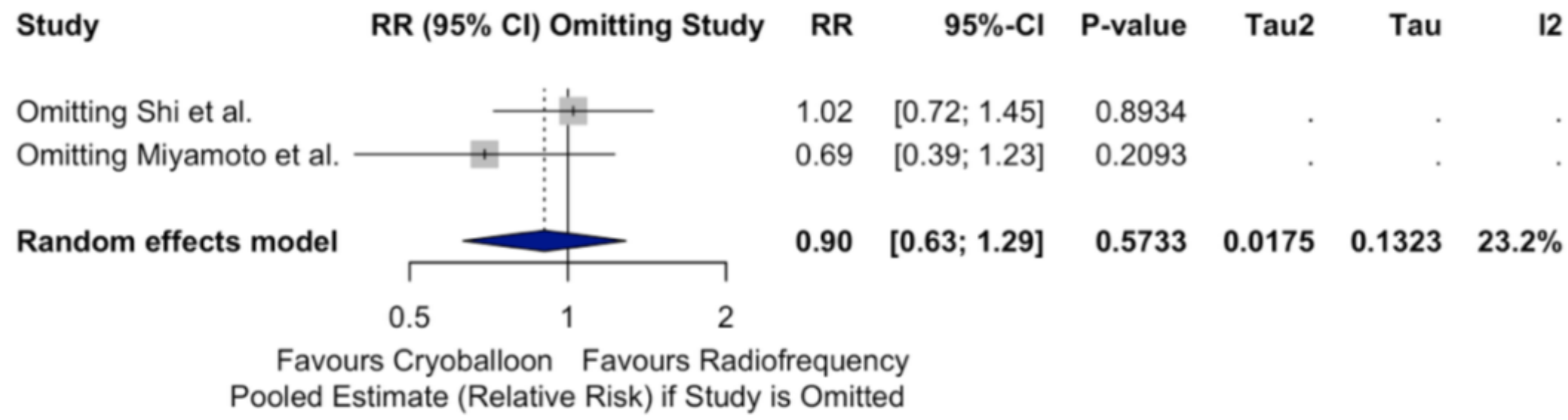

Supplementary Figure S3: Leave-one-out sensitivity analysis for freedom from AT/AFL.

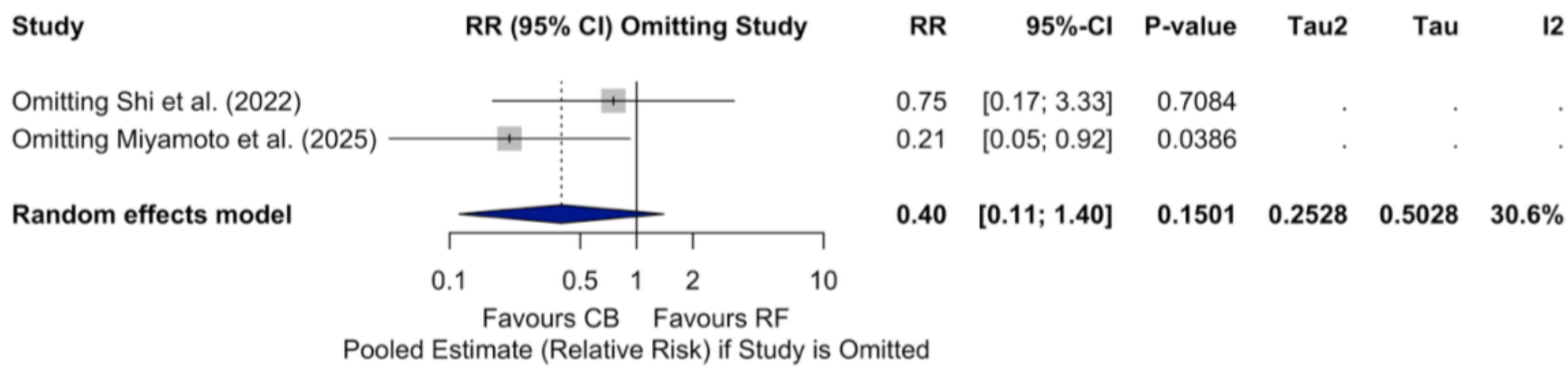

Supplementary Figure S4: Leave-one-out sensitivity analysis for procedural time.

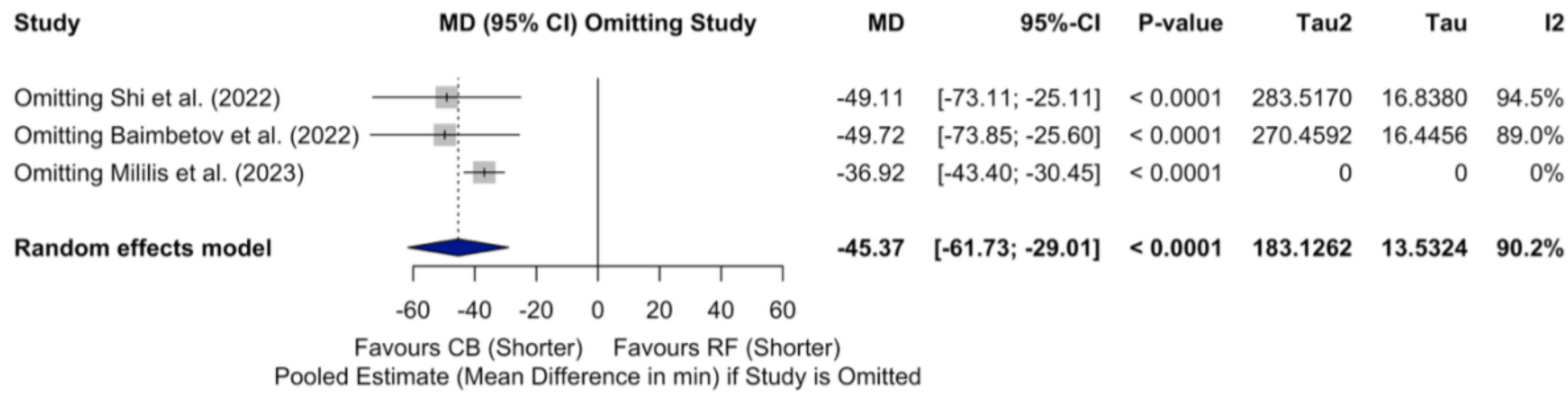

Supplementary Figure S5: Leave-one-out sensitivity analysis for fluoroscopy time.

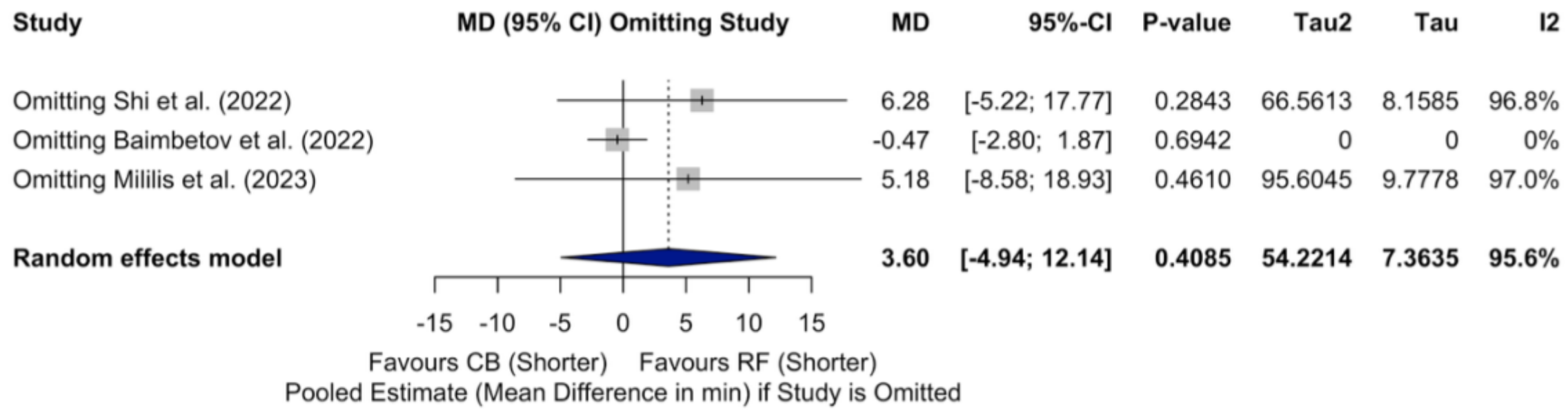

Supplementary Figure S6: Leave-one-out sensitivity analysis for repeat ablation.

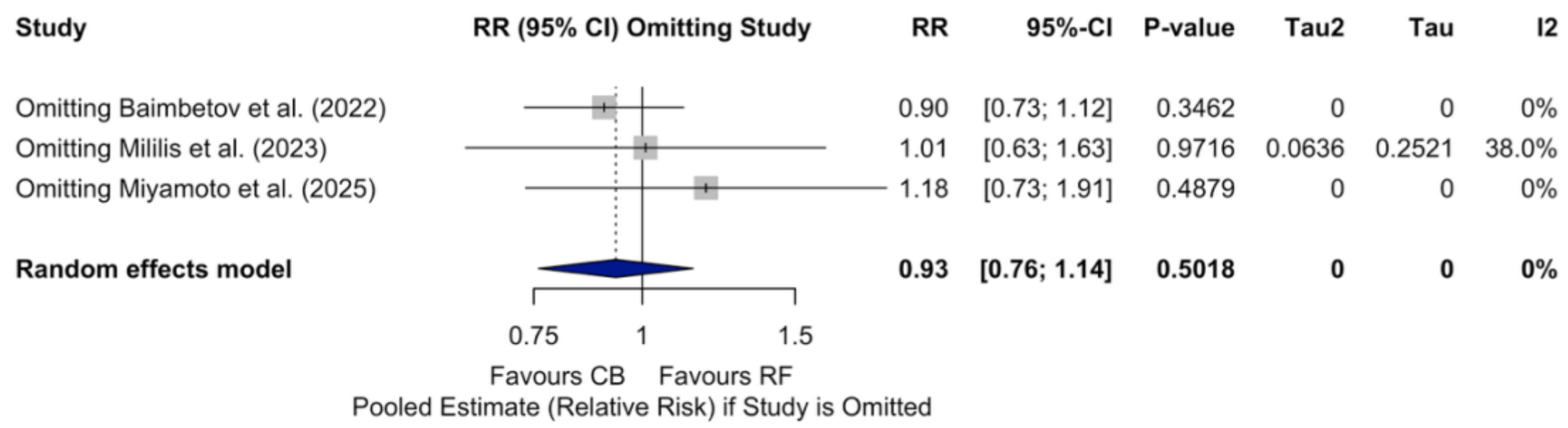

Supplementary Figure S7: Leave-one-out sensitivity analysis for any complications.

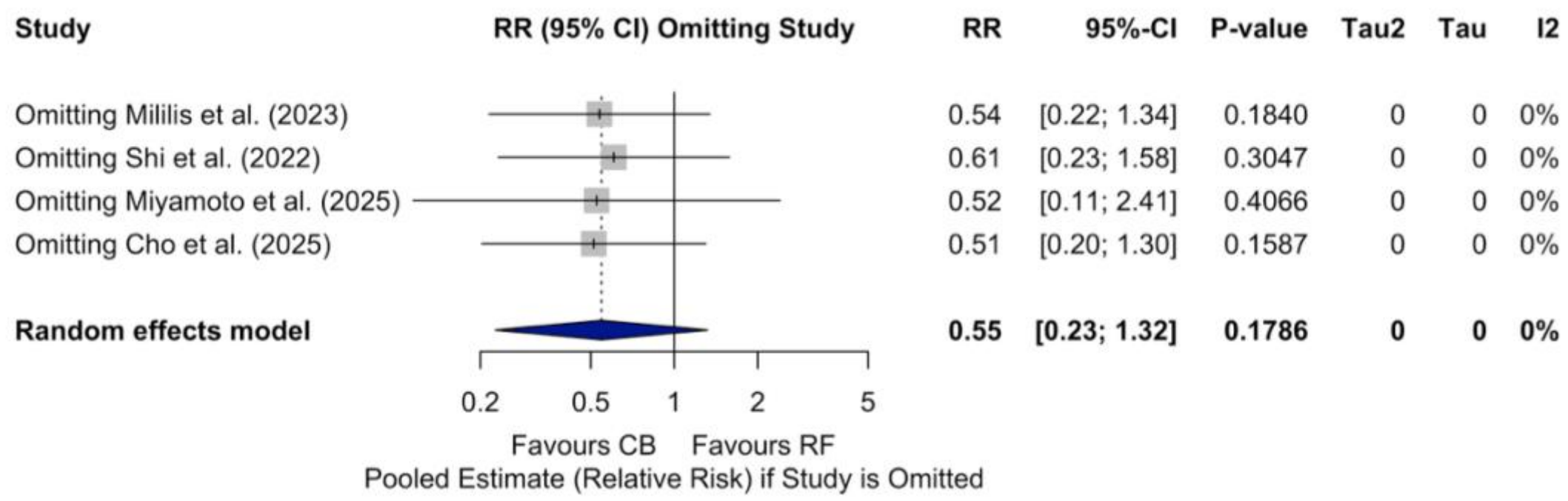

Supplementary Figure S8: Funnel plot for freedom from any arrhythmia at 12 months.

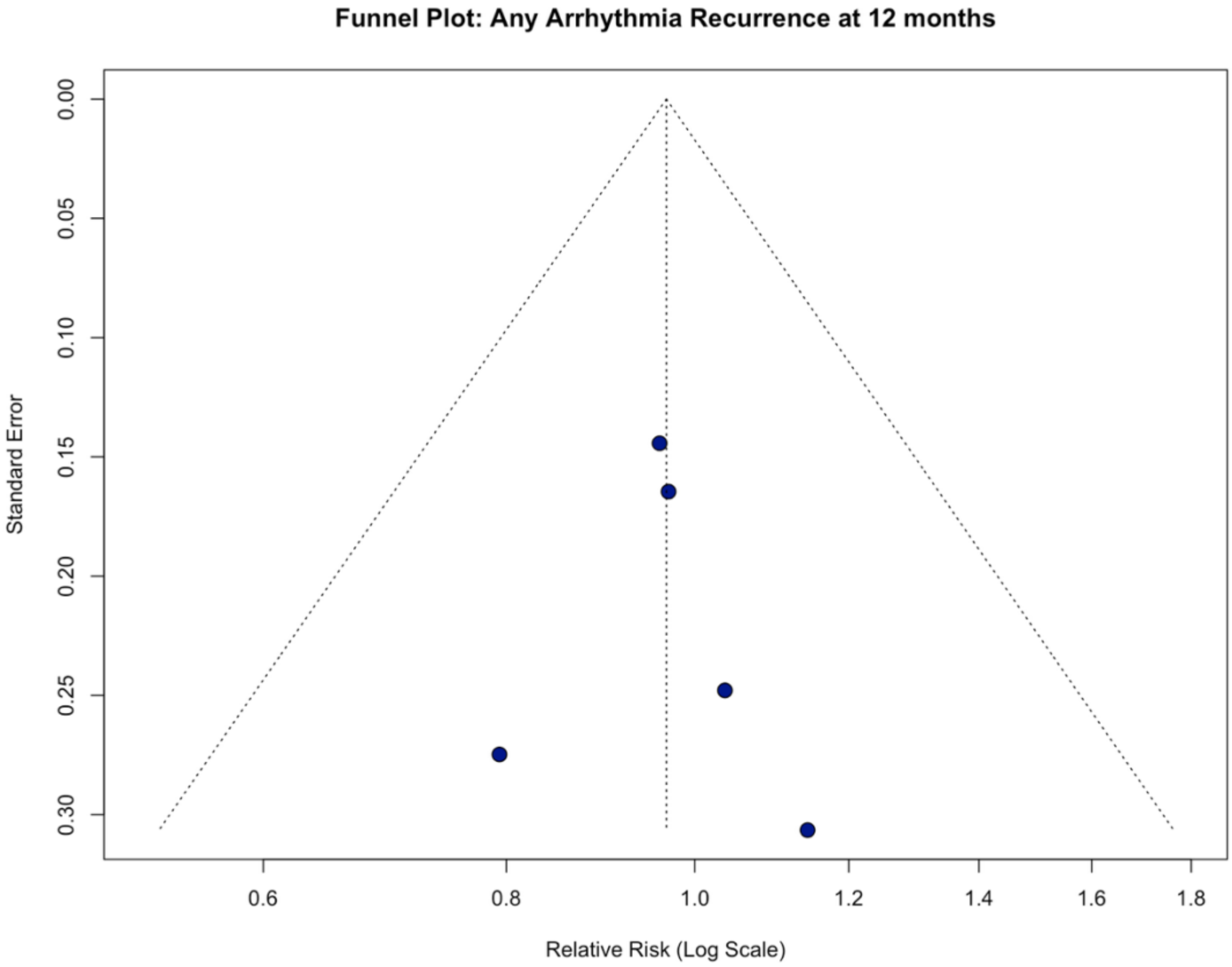

Supplementary Figure S9: Funnel plot for freedom from atrial fibrillation at 12 months.

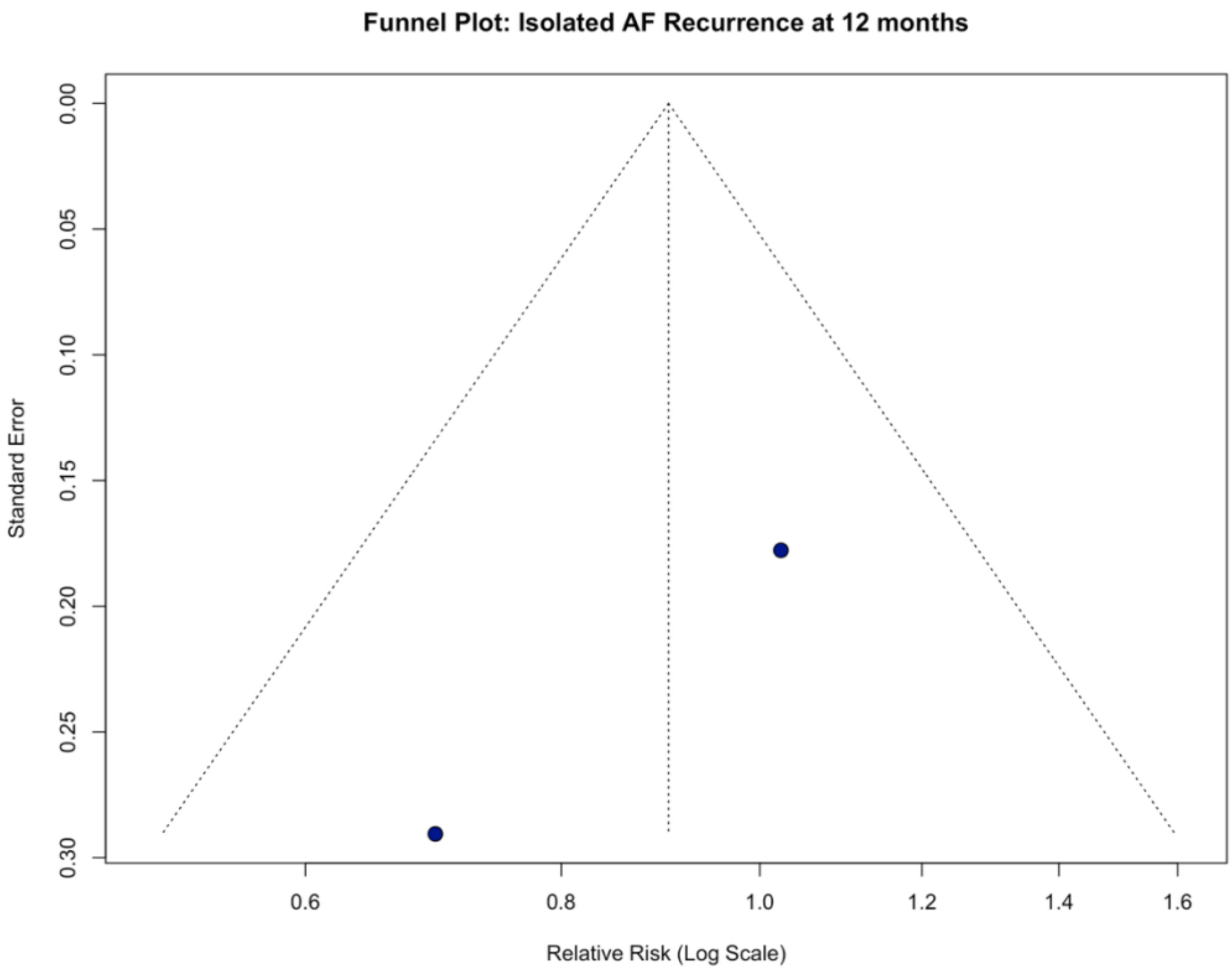

Supplementary Figure S10: Funnel plot for freedom from AT/AFL.

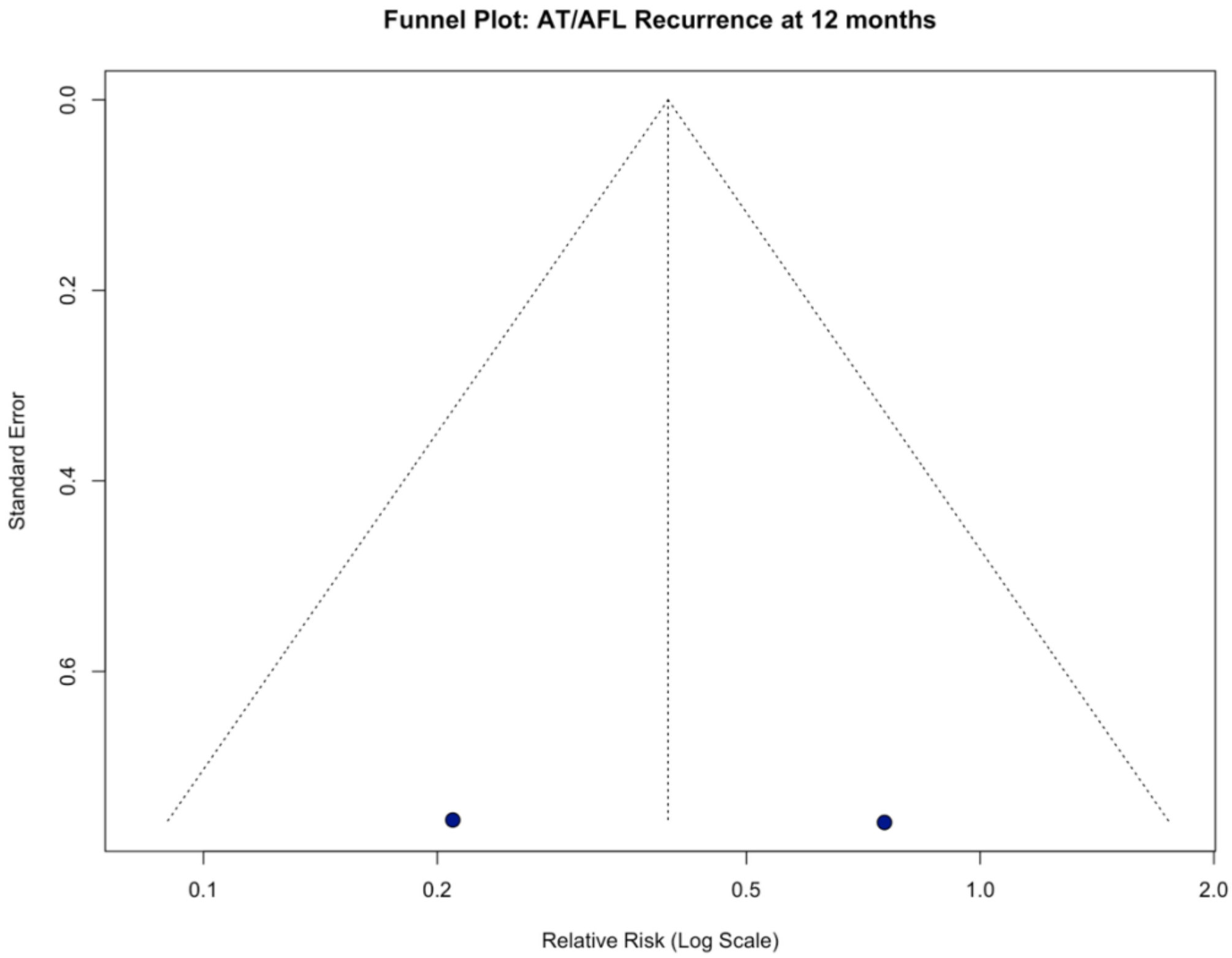

Supplement: Supplementary file 1 [file PACE-49-1692-s001.pdf]
